# Sufentanil postoperative analgesia reduce the increase of T helper *17* (Th*17*) cells and FoxP*3*^+^ regulatory T (Treg) cells in rat hepatocellular carcinoma surgical model: A randomised animal study

**DOI:** 10.1186/s12871-020-01129-0

**Published:** 2020-08-26

**Authors:** Yanhua Peng, Jinfeng Yang, Duo Guo, Chumei Zheng, Huiping Sun, Qinya Zhang, Shuangfa Zou, Yanping Zhang, Ke Luo, Keith A. Candiotti

**Affiliations:** 1 Department of Anesthesiology, Hunan Cancer Hospital, The Affiliated Cancer Hospital of Xiangya School of Medicine, Central South University, Changsha, 410013 Hunan China; 2 Department of Anesthesiology, Perioperative Medicine and Pain Management, University of Miami-Miller School of Medicine, Miami, FL 33136 USA

**Keywords:** Sufentanil, Postoperative analgesia, Th17, Treg

## Abstract

**Background:**

Surgery-related pain and opioids might exacerbate immune defenses in immunocompromised cancer patients which might affect postoperativd overall survival. Sufentanil is a good postoperative pain control drug,the present study aimed to figure out whether it effect T cell immunity in rat hepatocellular carcinoma surgical model.

**Methods:**

A rat hepatocellular carcinoma (HCC) models was established by N-nitrosodiethylamine. Forty-eight of them were randomly divided into 3 equal groups: surgery without postoperative analgesia (Group C), surgery with morphine postoperative analgesia (Group M), surgery with sufentanil postoperative analgesia (Group S). Each animal underwent a standard left hepatolobectomy, and intraperitoneally implanted with osmotic minipumps filled with sufentanil, morphine or normal saline according to the different group. The food and water consumptions, body weight changes, locomotor activity and mechanical pain threshold (MPT) were observed. The ratio of CD4^+^/CD8^+^, proportions of Th1, Th2, Th17 and Treg cells in blood were detected using flow cytometry. The liver function and the rats’ survival situation of each group were observed.

**Results:**

The food and water consumption, locomotor activity and MPT of group C declined than those of group S and M on d1, d2, d3 (*P < 0.05*). The CD4^+^/CD8^+^ ratio and the proportion of Th1 cells were significantly higher while the proportion of Th2, Th17 and Treg cells were significantly lower in group S and group M compared with group C. The rats of group S have higher CD4^+^/CD8^+^ ratio on d3, while lower proportion of Treg cells on d7 compared with group M. The plasma ALT and AST values in group C were significantly higher than that of group S and group M on both d3 and d7. There were not significant differences in mortality rate between 3 groups.

**Conclusions:**

Sufentanil and morphine postoperative analgesia in HCC rats accepted hepatectomy could relieve postoperative pain, promote the recovery of liver function after surgery, alleviate the immunosuppressive effect of pain. Furthermore, Compared to morphine, sufentanil might have a slighter effect on CD4^+^/CD8^+^ ratio and Treg frequencies. Therefore, sufentanil postoperative analgesia is better than morphine in HCC hepatectomy rats.

## Background

Hepatocellular carcinoma (HCC) is one of the most common malignant tumors, characteristic of relatively poor overall survival and increasing morbidity and mortality, which is reportedly the third cancer-related mortality worldwide [1, 2]. Surgery-related pain and opioid analgesics are factors known to adversely affect the anti-tumor immune defenses which may promote tumor growth and metastasis [3]. In view of the growing interest in the immune system in control of neoplasia, further efforts toward the discovery of a good analgesia agent for postoperative pain treatment with a reduced impaction on immunity are urgently needed.

The helper T cells were mainly divided into T helper 1(Th1), Th2, Th9, Th17, Th21, T follicular helper (Thf) and regulatory T (Treg) cells according to the function and phenotype [4, 5]. Among them, Th1, Th2, Th17 and Treg cells are more concerned in tumor immunity. Th17 cells could increase tumor progression by activating angiogenesis and immunosuppressive activities [6, 7]. Treg cells might inhibit the tumor-specific T cell-mediated immune response and have been observed increased quantity in tumor tissues or peripheral blood of patients or animal models with gastric cancer [8], ovarian cancer [9], breast cancer [10] and hepatocellular carcinoma [11].

Immune cells express appropriate receptors such as the μ receptor and toll-like receptor. Opioids modulate the immune system by binding to the μ receptor [12]. Sufentanil has a higher affinity to μ1-opioid receptor which has the closest relationship with analgesia than morphine, but the selectivity for binding to μ2 receptor is opposite which is related to adverse effects such as nausea, vomiting, respiratory depression, urinary retention, and itching, so sufentanil has stronger analgesic effect than morphine, and adverse effects are weaker than morphine [13].

The results of opioid-induced immunomodulation are conflicting in experimental and human studies. Previous studies manifested that morphine could decrease the expressions of peripheral T lymphocytes (CD3^+^, CD4^+^, CD8^+^) and natural killer cells (CD3^+^, CD56^+^) in vivo [14] and could increase the ratio of CD4^+^/CD8^+^ T cells and Treg populations in vitro [15]. The Epidural postoperative analgesia with ropivacaine plus sufentanil significantly decreased Blymphocytes, T-helper cells and Natural killer cells compared with patient-controlled IV analgesia (PCIA) with morphine in patients after major spine surgery [16]. However, little or nothing is known concerning the effect of sufentanil postoperative analgesia on Th17 and Treg cells. The primary purpose of this study was to observe the effects of sufentanil and morphine postoperative analgesia on immunity through analysis of CD4^+^/CD8^+^ ratio, proportion of Th1, Th2, Th17 and Treg cells using flow cytometry, and the secondary target was liver function changes and mortality in HCC rats undergoing left hepatolobectomy.

## Methods

### Ethics

All animal procedures were approved (Permit Number: 2015001) by the Institutional Animal Care and Use Committees of Hunan Cancer Hospital, Changsha, China on 27 March 2015, and were performed in strict accordance with recommendations of the Guide to the Care and Use of Laboratory Animals of the National Institutes of Health.

### Animals

Eighty male Sprague-Dawley rats (100 ± 20 g; Center of Experimental Animals of Hunan Cancer Hospital, Hunan, China) were used in this experiment. Rats were housed under controlled conditions with a temperature of 25 ± 2 °C, relative humidity of 60 ± 10*%*, room air changes of 12–18 times/h and a 12 h light/dark cycle and were acclimated for 7 days before experiments. They were allowed free access to food and water.

### Experimental protocol

Eighty Sprague-Dawley rats were intraperitoneal administrated with 0.19*%* N-nitrosodiethylamine (DENA, Sigma Aldrich, USA)(50 mgkg^− 1^) every 3 days for a total of 16 weeks to make HCC models [17]. After 16 weeks, 58 of these rats were successfully modeled, 48 HCC rats were randomly selected and stochasticly assigned to 3 groups by digital random method(*n* = 16): surgery without postoperative analgesia (Control, Group C), surgery with morphine postoperative analgesia (Group M), surgery with sufentanil postoperative analgesia (Group S). All animals underwent a standard left hepatolobectomy under 2–3*%* isoflurane anesthesia. Rats’ abdominal region was shaved and thoroughly cleaned with complex iodine. A 2 cm midline incision was made in the abdomen. After reaching the abdomen cavity, the left lateral leaf of the liver was exposed, and the left leaves were ligated from the root and excised. A implanted osmotic minipumps (volume 2 ml, pump speed 10μlh^− 1^ for 72 h, Alzet, USA) for postoperative analgesia was placed in the abdominal cavity, which is filled with morphine of 0.25 mgKg^− 1^h^− 1^for 72 h in Group M [18], sufentanil of 0.25ugKg^− 1^ h^− 1^ for 72 h in Group S (the dose of sufentanil was calculated in accordance with its analgesic potency in comparison to morphine), or 0.9*%* saline 10ul h^− 1^ for 72 h in Group C. Finally, the muscle and skin were closed with sterile sutures. During surgery, the rats’ temperatures were maintained using a thermal insulation blanket.

We measured the following parameters in each operated rat on 1 day before surgery (d0), the first, second and third day after surgery(d1, d2, d3): Food and water consumption, body weight changes. The locomotor activity was surveyed using open field test [19]. Mechanical pain threshold (MPT) comprehensively evaluated using standard von Frey monofilaments [20]. We randomly sacrificed four rats per group on 1 day before surgery (d0), six rats on the third day after surgery (d3), and all of the remaining rats on the seventh day after surgery (d7) to collect blood samples by cardiac puncturing method, and all the rats were euthanized by the method of cercical vertebra decoupling under anesthesia after collecting blood samples. The level of cluster of CD4^+^, CD8^+^, Th1, Th2, Th17 and Treg cells in blood were detected to assess immune function using flow cytometry on d0, d3 and d7. The serum alanine aminotransferase (ALT) and aspartate transaminase (AST) were measured to assess liver function at the same time point. The rats’ survival situation of each group left after 7 days of surgery were observed.

### Locomotor activity—open field test

Rats were individually exposed to the same open field (100 cm × 100 cm) for *5* min trials with an interval of 30 min between each trial. The open field behavior was videotaped using a camera that was placed above the arena. The videos were subsequently analyzed digitally using EthoVisionXT (Noldus, The Netherlands). Parameters measured were the total distance traveled throughout the arena.

### Mechanical pain threshold (MPT)

Rats were placed in test cages prior to the experiment and allowed to fully acclimate to the environment for 3 h. A 0.1 to 12 g single fiber test needle was used to stimulate the position of the rat’s abdominal incision about 0.5 cm perpendicular to the skin surface until the filament was slightly curved in an S shape for 5–6 s. The MPT for this region was measured using the Chaplan up-down method [21]. If the rat appears to be licking or scratching the stimulated area during the stimulation time or removing the von Frey filament, or a sudden withdrawal or jump occurs, it is recorded as a positive behavioral response.

### Assessment of liver function

Blood samples were collected and sera were obtained by centrifugation in low temperature on d0, d3, d7. Serum AST and ALT were measured using the modified Jaffe rate reaction in the clinical laboratory of The Hunan Cancer Hospital, Changsha, China.

### Flow cytometry

Fresh heparinized blood samples of rats were collected on d*0*, d*3*, d*7*. Then Peripheral Blood Mononuclear Cells (PBMCs) were isolated from blood by standard density gradient separation using Ficoll density gradient (TBD Science, China). Each specimen is divided into five equal parts in testing CD4^+^, CD8^+^, Th1, Th2, Th17 and Treg cells. Isolated cells were washed three times with phosphate buffer saline and used for flow cytometry. A total of 1 × 10^5^ PBMCs prepared for were acquired for each sample. Each sample was surface stained with CD3-PE, CD4-FITC plus PE-Cy7-labeled anti-rat CD8 (BD Bioscience, USA) to detect CD4^+^T cells, CD8^+^T cells at room temperature for 15 min (avoid light). The subsets detection needed analyze CD4 combined with specific cytokines such as CD4^+^IFN-γ^+^ for Th1, CD4^+^IL-4^+^for Th2, and CD4^+^IL-17^+^ for Th17. For the Th1, Th2, Th17, samples were surface stained with CD4-FITC at room temperature for 15 min (avoid light), and subsequently stimulated for the intracellular cytokines with PE-labeled anti-rat IFN-r, PE-labeled anti-rat IL-4, PE-labeled anti-rat IL-17A(BD Bioscience, USA) respectively according to the manufacturer’s instructions. The CD4^+^Foxp3^+^ phenotype was recommended for identifying the Treg. Though, samples were surface stained with CD4-FITC at room temperature for 15 min (avoid light), and subsequently intracellularly stained with a PE anti-rat Foxp*3* staining kit (BD Bioscience, USA) without stimulated according to the manufacturer’s instructions. Cells were detected by flow cytometry using a FACSCalibur (BD Bioscience, USA), and data were analyzed by FlowJo VX (Treestar, USA).

### Statistical analysis

Data are shown as mean ± SD for normally distributed data. Probability values< 0.05 were considered statistically significant. Then the data was transferred to the computer using SPSS Statistics 25.0(IBM, USA), normally distributed data were analyzed by using a one-way ANOVA followed by a post hoc S-N-K test (Equal variances assumed) and Tamhane T2 test (Equal variances not assumed) to compare the three groups at each time point. The descriptive findings were compared using Fisher’s exact test with *P < 0.0001*.

## Results

**Fig. 1:**
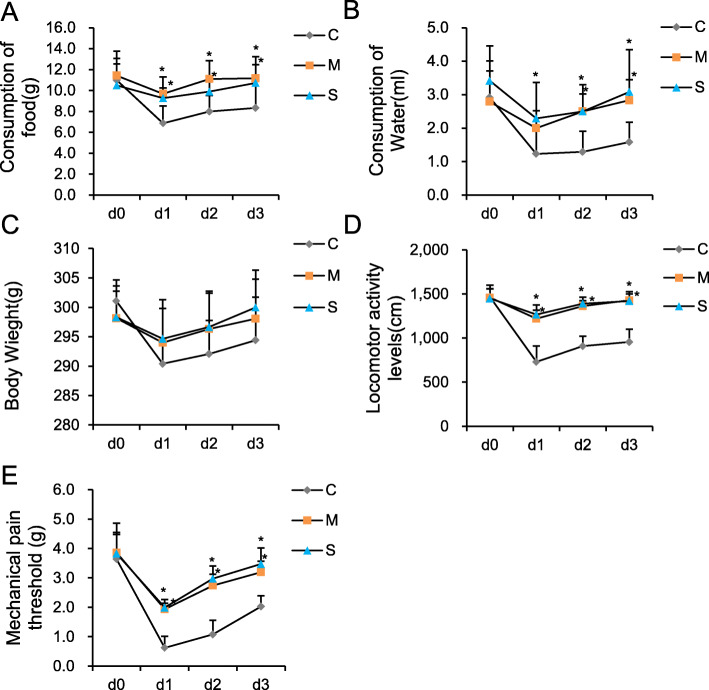
The consumption of food and water, body weight, locomotor activity and the mechanical pain threshold for each of the 3 groups. **a** Statistical analysis of the consumption of food in each of the 3 groups. **b** Statistical analysis of the consumption of water in each of the 3 groups. **c** Statistical analysis of body weight in each of the 3 groups. **d** Statistical analysis of locomotor activity in each of the 3 groups. **e** Statistical analysis of the mechanical pain threshold in each of the 3 groups. The consumption of food and water, locomotor activity and mechanical pain threshold of Group C decline to a significantly lower degree than those of Group S and Group M on d1, d2, d3. *P < 0.05 vs Group C. d0, base line. d1, d2 and d3, first, second and third day after surgery

**The food and water consumption, body weight, locomotor activity and the pain threshold in each group—**The food consumption, water consumption, locomotor activity and MPT of Group C decline to a significantly lower degree than those of Group S and M on d1, d2, d3(*P* < 0.05). The food consumption, water consumption, locomotor activity and pain threshold of Group S were similar with that of Group M at each time point. There were no significant differences of the body weight between the three groups (Fig. 1).

**Fig. 2:**
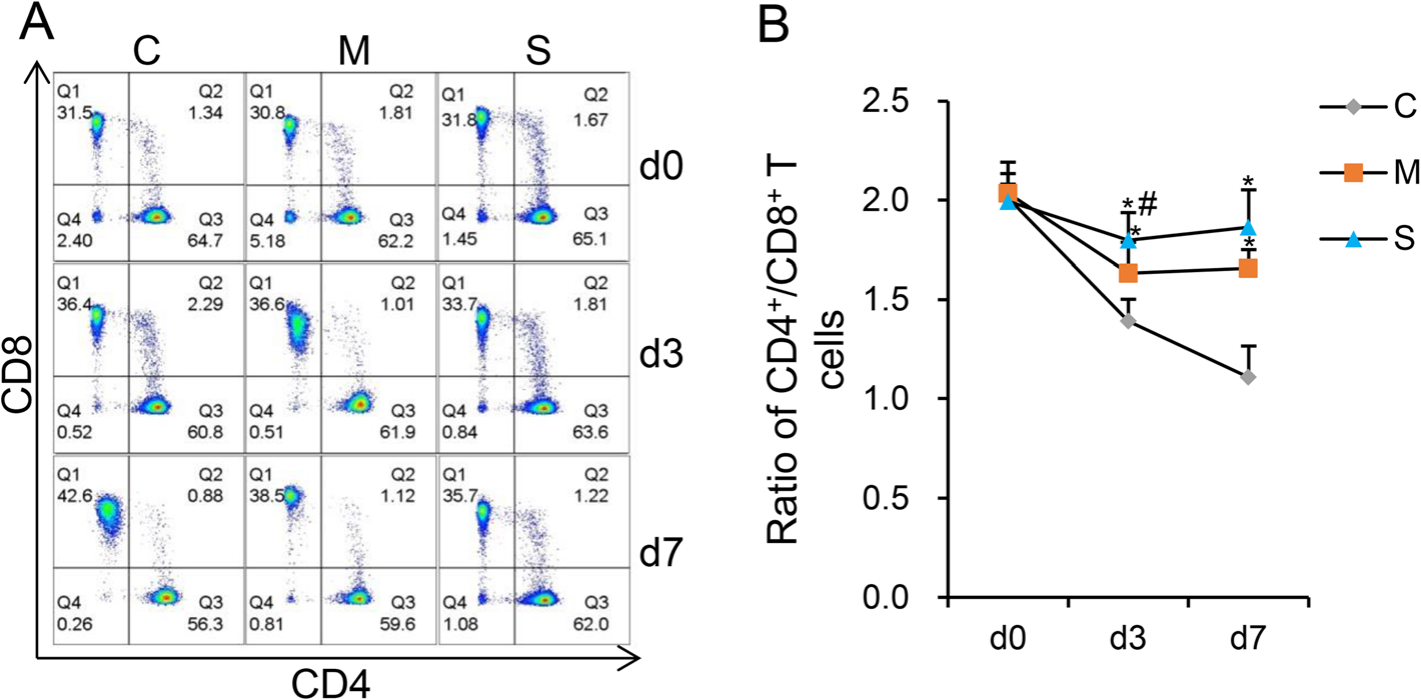
The CD4^+^/CD8^+^ ratio in each of the 3 groups. **a** Flow cytometric analysis of CD4^+^ and CD8^+^ cells levels in the 3 groups. **b** Statistical analysis of CD4^+^/CD8^+^ ratio for each of the 3 groups. The ratio of CD4^+^/CD8^+^ in Group S and Group M was significantly higher than was noted in Group C on d3 and d7. The Group S had a higher ratio of CD4^+^/CD8^+^ than was noted in Group M on d3. *P < 0.05 vs Group C. # *P* < 0.05 Group S vs Group M. d0, base line, d3 and d7, third and 7th day after surgery

**CD4**^**+**^**/CD8**^**+**^**ratio in blood of each group—**Fig. 2. A shows the flow cytometric analysis of CD4^**+**^ and CD8^**+**^ cells. Figure 2. B shows the statistical analysis of CD4^**+**^/CD8^**+**^ ratio. The CD4^**+**^/CD8^**+**^ ratio of Group S and Group M were significantly higher than that of Group C on d3 and d7 (*P < 0.05*). The CD4+/CD8+ ratio of Group S was significantly higher than that of Group M on d3 (*P < 0.05*).

**Fig. 3:**
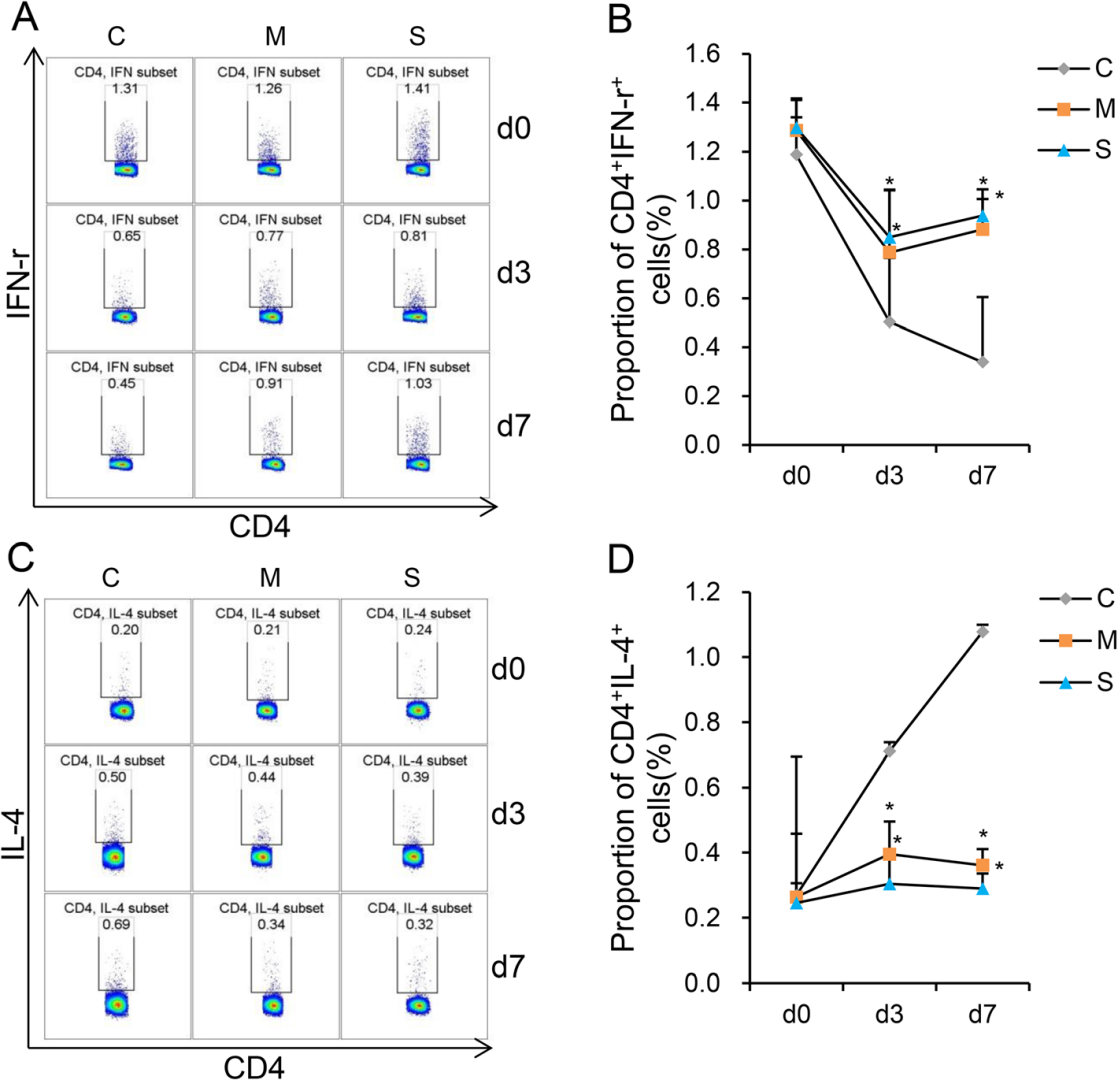
Th1 and Th2 cells levels in the 3 groups. (**a** and **c**) Flow cytometric analysis of Th1 and Th2 cells levels in the 3 groups. (**b** and **d**) Statistical analysis of the proportion of Th1 and Th2 cells. The proportion of Th1 cells in Group S and Group M was significantly higher than was noted in Group C on d3 and d7. The proportion of Th2 cells in Group S and Group M, however, was significantly lower than was noted in Group C on d3 and d7. *P < 0.05 vs Group C. d0, base line; d3 and d7, third and 7th day after surgery

**The proportion of Th1 and Th2 cellsin blood of each group—**Fig. 3. A and C shows the flow cytometric analysis of Th1 (CD4^+^IFN-γ^+^) and Th2 (CD4^+^IL4^+^) cells. Figure 3. B and D shows the statistical analysis of Th1 and Th2 cells. The proportion of Th1 cells of Group S and Group M were significantly higher than that of Group C on d3 and d7 (*P < 0.05,* Fig. 3. B). The proportion of Th2 cells of Group S and Group M were significantly lower than that of Group C on d3 and d7 (*P < 0.05,* Fig. 3. D). There were no statistically significant differences in proportion of Th1 and Th2 cells between Group S and Group M on d3 and d7 (Fig. 3. B and D).

**Fig. 4:**
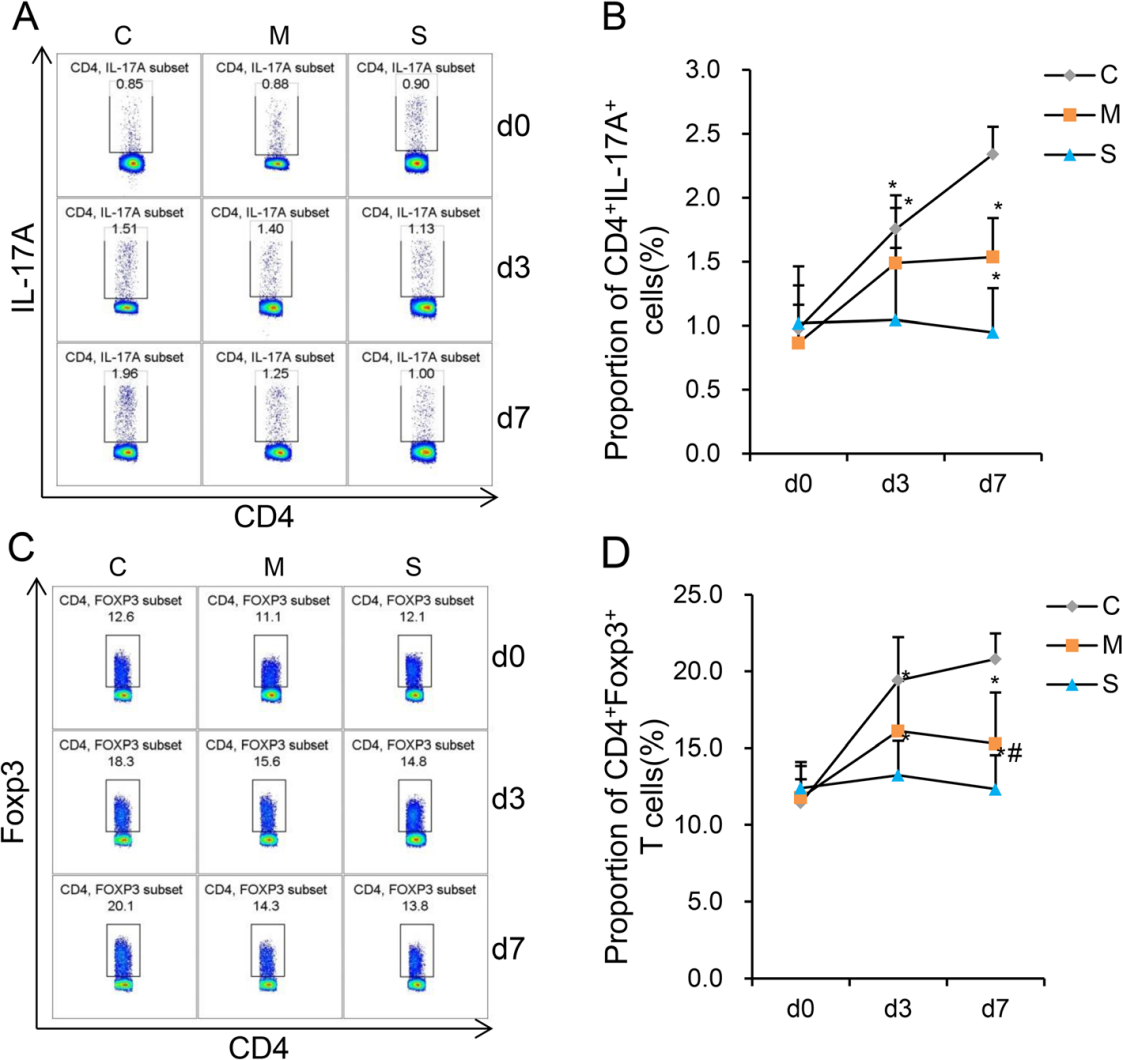
Th17 and Treg cells levels in the 3 groups. (**a** and **c**) Flow cytometric analysis of Th17 and Treg cells levels in the 3 groups. (**b** and **d**) Statistical analysis of the proportion of Th17 and Treg cells. The proportion of Th17 and Treg cells in Group S and Group M was significantly lower than in Group C on d3 and d7. Compared to Group M, the proportion of Treg cells in Group S was significantly lower on d7. *P < 0.05 vs Group C. d0, base line; d3 and d7, third and 7th day after surgery

**The proportion Th17 and Treg cells in blood of each group**—Fig. 4. A and C shows the flow cytometric analysis of Th17 (CD4^+^IL17-A^+^) cells and Treg (CD4^+^Foxp3^+^) cells. Figure 4. B and D shows the statistical analysis of Th17 and Treg cells. Rats showed lower proportion of Th17 and Treg cells in Group S and Group M than group C (*p < 0.05,* Fig. 4. B and D). There were no statistically significant differences in proportion of Th17 cells between Group S and Group M on d3 and d7, however, the proportion of Treg cells in Group S was significantly lower in comparison to Group M on d7 (*p < 0.05,* Fig. 4. D).

**Fig. 5:**
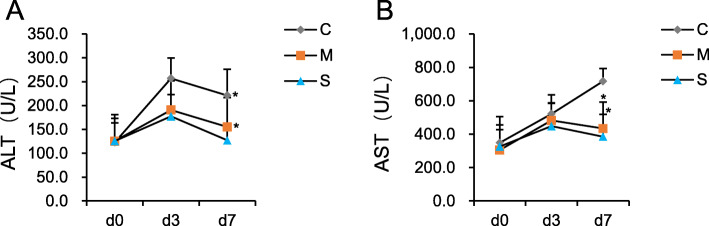
Liver enzymes for each of the 3 groups. The ALT(**a**) and AST(**b**) levels in the Group S and Group M were statistically higher than those in the Group C at d7. *P < 0.05 vs Group C. # *P* < 0.05 Group S vs Group M. d0, base line, d3 and d7, third and 7th day after surgery

**The liver function in each group after surgery—**A significant increase of ALT and AST levels was observed in group C in comparison to Group S and Group M on d7(*p* < *0.05*). But no statistically significant difference was observed between Group S and Group M (Fig. 5. A and B).

**The survival situation—**Though we did not find statistically significant differences in mortality rate between postoperative analgesia rats and without analgesia rats (*P* = *0.245*, Fisher’s Exact Test). We did observe that two rats of Group C died respectively on fourth and fifth day after surgery, one rat of Group M died on sixth day after surgery, and no rat died in Group S.

## Discussion

This study found that sufentanil and morphine postoperative analgesia rats have higher CD4^+^/CD8^+^ ratio, Th1 cells level while lower Th2, Th17 and Treg cells levels compared with that without postoperative analgesia. Sufentanil postoperative analgesia rats have higher CD4^+^/CD8^+^ratio on the third day after surgery while lower Treg cells level on the 7th day after surgery in comparison to morphine postoperative analgesia rats.

Acute postoperative pain can activate the hypothalamic–pituitary–adrenal axis, affect metabolism and cause neuroendocrine changes, which are strongly associated with postoperative outcome [12, 22, 23]. Postoperative pain relief can reduce surgery-associated cardiac, pulmonary, metabolic complications, and improve immune status which may improve the postoperative outcome [24].

There is such a view that the interaction between CD4^+^ and CD8^+^ T lymphocyte mediates the control of tumor growth [25]. In a clinical study, the 5-year survival rate of cervical cancer patients with high CD4^+^/ CD8^+^ ratio was higher than that of patients with low CD4^+^/ CD8^+^ ratio, increasing the CD4^+^/ CD8^+^ ratio can slow the progression of cervical cancer and improve its prognosis [26]. It is generally believed that Th1 enhances tumor immune surveillance of tumor while Th2 associated with the tumor immune evasion can suppress the function of Th1 cells [27]. Th17 cells in peripheral blood are positively correlated with the progression of liver cancer [28]. Treg cells play a vital role in maintaining immunological homeostasis and exert major immunosuppressive activity [29]. A recent study has indicated that the percentages of CD4^+^CD25^+^FOXP3^+^Treg cells and CD4^+^IL-17^+^Th17 cells were significantly higher in HCC patients than in the healthy individuals; Moreover, the increased percentages of Treg and Th17 cells were closely related to the tumor stage and tumor size of HCC [11]. Most published research have found that post-operative opioids inhibit cell-mediated immunity and promote tumor metastasis for both human and mouse [3]. Some patients choose to tolerate pain because of concerns about the immunosuppressive effect of analgesics. Is this appropriate? In our study, the CD4^+^/CD8^+^ratio, proportion of Th1 cells were obviously higher while proportion of Th2, Th17 and Treg cells were significantly lower in group S and group M compared with group C. Therefore, it seems that sufentanil and morphine postoperative analgesia can alleviate the immunosuppressive effect of HCC surgery and postoperative pain and is more conducive to postoperative recovery than tolerating pain.

In terms of analgesic effect, both of sufentanil and morphine can control postoperative pain. Which is better for sufentanil and *morphine* postoperative analgesia? Previous studies have demonstrated that morphine affect the signal transportation of activated T cells, thereby inhibiting T-cell activation. Morphine increases the ratio of CD4^+^/CD8^+^and Treg cells populations [15, 30, 31], shifts the balance of Th1/Th2 cells toward Th2 cells [32, 33], while in vivo studies, the ratio of CD4^+^/CD8^+^cells, the proportion of Th1 and Th17 T cell were not changed with the administration of morphine [30]. Sufentanil increased the quantity of the Tregs to a greater degree than fentanyl when the culturing was conducted in vitro, while there was no significant difference between them in vivo [34]. In a clinical trial, total CD3^+^, CD4^+^, CD8^+^ cells and the ratio of CD4^+^/CD8^+^ cells in the sufentanil group were significant higher than that in the remifentanil group [35]. There are few direct comparative studies which involved in the effects of sufentanil and morphine on immunity. In our study, sufentanil and morphine have a similar effect on Th1, Th2, Th17 frequencies. Yet, the ratio of CD4^+^/CD8^+^ on d3 after surgery and the ratio of Treg cells on d7 after surgery in Group S is obviously less than that of Group S. This results indicate that sufentanil’s inhibition on CD4^+^ cells is lighter than morphine, but this inhibition may disappear with the withdrawal of the drugs. While the inhibition of sufentanil on Treg cells is less than that of morphine, but the inhibition of Treg cells may be manifested later. Recently, increasing studies have shown that there is a close positive correlation between recurrence and metastasis to the inhibition of immune system [36, 37]. Therefore, it seems that sufentanil is superior to morphine for postoperative anelgesia. The findings of the presented study provide help for the selection of postoperative analgesic drugs in clinic.

AST and ALT are important enzymes which represent liver cells function [38]. AST and ALT evidently increased can reflect severe liver cells necrosis. AST/ALT were the independent risk factors of overall survival [39]. Previous retrospective study indicated that hepatic cancer patients who underwent hepatectomy with higher ALT level had shorter mean recurrent interval than patients with lower ALT level [40]. In our study, we found that postoperative plasma ALT and AST values on the seventh day in Group S and Group M were significantly lower than in Group C. This suggests that postoperative analgesia can prevent liver function damage in HCC rats accepted hepatectomy.

There are some shortcomings in our experiment. First, we only measured the number of some T cell subsets without measuring important immune factors, such as TGF-beta, IL-6, IFN-gamma. Second, these rats’ long-term survival, metastasis rates were not observed. We will evaluate these results in the future.

## Conclusions

The current results have shown that sufentanil and morphine postoperative analgesia in HCC rats accepted hepatectomy can relieve postoperative pain, promote the recovery of liver function after surgery, alleviate the immunosuppressive effect of pain. Furthermore, Sufentanil postoperative analgesia is better than morphine resulted by the differences of CD4^+^/CD8^+^ ratio and Treg cells level after surgery.

## Data Availability

The data sets used and/or analyzed during the current study are available from the corresponding author on reasonable request.

## Abbreviations

ALT: represent serum alanine aminotransferase
AST: represent aspartate transaminase
DENA: represent N-nitrosodiethylamine
USA: represent United States of America
HCC: represent hepatocellular carcinoma
Th: represent T helper cell
Thf: represent T follicular helper
Treg: represent regulatory T cells
MPT: represent Mechanical pain threshold
PBMCs: represent Peripheral Blood Mononuclear Cells
